# Supplementary material for: Restoring South African subtropical succulent thicket using Portulacaria afra: root growth of cuttings differs depending on the harvest site during a drought
Source: PeerJ. 2024 Jun 28;12:e17471. doi: 10.7717/peerj.17471 (PMC11216190; doi:10.7717/peerj.17471)
Supplement: Supplemental Information 1 — The precipitation values are from the CHIRPS dataset and are shown here to compare potential precipitation differences amongst sites. We consider these satellite-derived estimated values higher than precipitation actually received in the field—e.g., a landowner’s rain gauge ∼3 km from population 8 only received 119 mm during the 12-month period prior to harvesting. Sites were sampled from three landscape positions: inland mountains (1, 2, 5, 6), inland lowlands (3, 4, 7, 8) and coastal hills (9, 10). [file peerj-12-17471-s001.docx]

|  | Precipitation (mm) in preceding months | | | |
| --- | --- | --- | --- | --- |
| Site | 3 | 6 | 9 | 12 |
| 1 | 44 | 99 | 231 | 372 |
| 2 | 44 | 117 | 260 | 393 |
| 3 | 23 | 96 | 211 | 331 |
| 4 | 26 | 98 | 222 | 336 |
| 5 | 35 | 136 | 253 | 420 |
| 6 | 67 | 158 | 299 | 462 |
| 7 | 54 | 142 | 263 | 439 |
| 8 | 54 | 142 | 263 | 439 |
| 9 | 72 | 166 | 292 | 486 |
| 10 | 87 | 215 | 343 | 562 |
