# Supplementary material for: Restoring South African subtropical succulent thicket using Portulacaria afra: root growth of cuttings differs depending on the harvest site during a drought
Source: PeerJ. 2024 Jun 28;12:e17471. doi: 10.7717/peerj.17471 (PMC11216190; doi:10.7717/peerj.17471)
Supplement: Supplemental Information 2 — Significant differences within sampling events amongst source populations are shown using dissimilar superscripts (tests for significant differences were conducted using Kruskal–Wallis and post-hoc Dunn tests across populations for each harvest week). [file peerj-12-17471-s002.docx]

|  | Percentage Rooted Cuttings (%) Across Sites | | | | | | | | | |  |
| --- | --- | --- | --- | --- | --- | --- | --- | --- | --- | --- | --- |
| Sampling event | 1 | 2 | 3 | 4 | 5 | 6 | 7 | 8 | 9 | 10 | Kruskal-Wallis Test |
| 20 | 47±14^ab^ | 67±26^ab^ | 20±18^ab^ | 10±15^a^ | 17±19^ab^ | 91±13^b^ | 17±17^ab^ | 13±14^a^ | 83±20^ab^ | 93±9^b^ | 𝝌^2^_(9)_=38.0, p<0.0001 |
| 27 | 73±15^abc^ | 97±7^ab^ | 43±25^ac^ | 27±19^c^ | 54±16^abc^ | 100±0^b^ | 60±25^abc^ | 53±22^abc^ | 87±14^abc^ | 100±0^b^ | 𝝌^2^_(9)_=36.8, p<0.0001 |
| 35 | 78±16^abc^ | 97±7^ab^ | 55±39^abc^ | 31±30^c^ | 76±21^abc^ | 100±0^a^ | 72±26^abc^ | 45±21^bc^ | 96±9^abc^ | 97±7^ab^ | 𝝌^2^_(9)_=30.9, p<0.001 |
| 42 | 90±9^ab^ | 93±9^ab^ | 67±42^ab^ | 43±25^a^ | 83±14^ab^ | 100±0^b^ | 90±9^ab^ | 67±31^ab^ | 100±0^b^ | 100±0^b^ | 𝝌^2^_(9)_=27.2, p=0.001 |
| 48 | 97±7^a^ | 97±7^a^ | 63±32^a^ | 50±33^a^ | 79±25^a^ | 100±0^a^ | 93±15^a^ | 63±30^a^ | 93±15^a^ | 100±0^a^ | 𝝌^2^_(9)_=25.9, p=0.002 |
| 56 | 100±0^a^ | 100±0^a^ | 70±34^ab^ | 60±35^b^ | 96±8^ab^ | 100±0^a^ | 100±0^a^ | 67±26^ab^ | 90±22^ab^ | 100±0^a^ | 𝝌^2^_(9)_=29.7, p<0.001 |
| 103 | 93±15^ab^ | 100±0^a^ | 67±39^ab^ | 73±35^ab^ | 88±25^ab^ | 100±0^a^ | 97±7^ab^ | 57±19^b^ | 93±15^ab^ | 100±0^a^ | 𝝌^2^_(9)_=25.1, p=0.003 |
