# Supplementary material for: Restoring South African subtropical succulent thicket using Portulacaria afra: root growth of cuttings differs depending on the harvest site during a drought
Source: PeerJ. 2024 Jun 28;12:e17471. doi: 10.7717/peerj.17471 (PMC11216190; doi:10.7717/peerj.17471)
Supplement: Supplemental Information 3 [file peerj-12-17471-s003.docx]

| Sampling Event | Non-normal sites excluded | All sites |
| --- | --- | --- |
| 35 | F_8,35_=4.32, p=0.001 | F_9,39_=4.38, p=0.0005 |
| 42 | F_7,32_=2.92, p=0.018 | F_9,39_=6.08, p<0.0001 |
| 48 | F_7,31_=10.60, p<0.0001 | F_9,39_=9.18, p<0.0001 |
| 56 | * | F_9,39_=11.9, p<0.0001 |
| 103 | F_8,35_=6.35, p<0.0001 | F_9,39_=8.20, p<0.0001 |

* All sites had a normal distribution for mean dry root mass.
