# Supplementary figures and images for: Restoring South African subtropical succulent thicket using Portulacaria afra: root growth of cuttings differs depending on the harvest site during a drought
Source: PeerJ. 2024 Jun 28;12:e17471. doi: 10.7717/peerj.17471 (PMC11216190; doi:10.7717/peerj.17471)

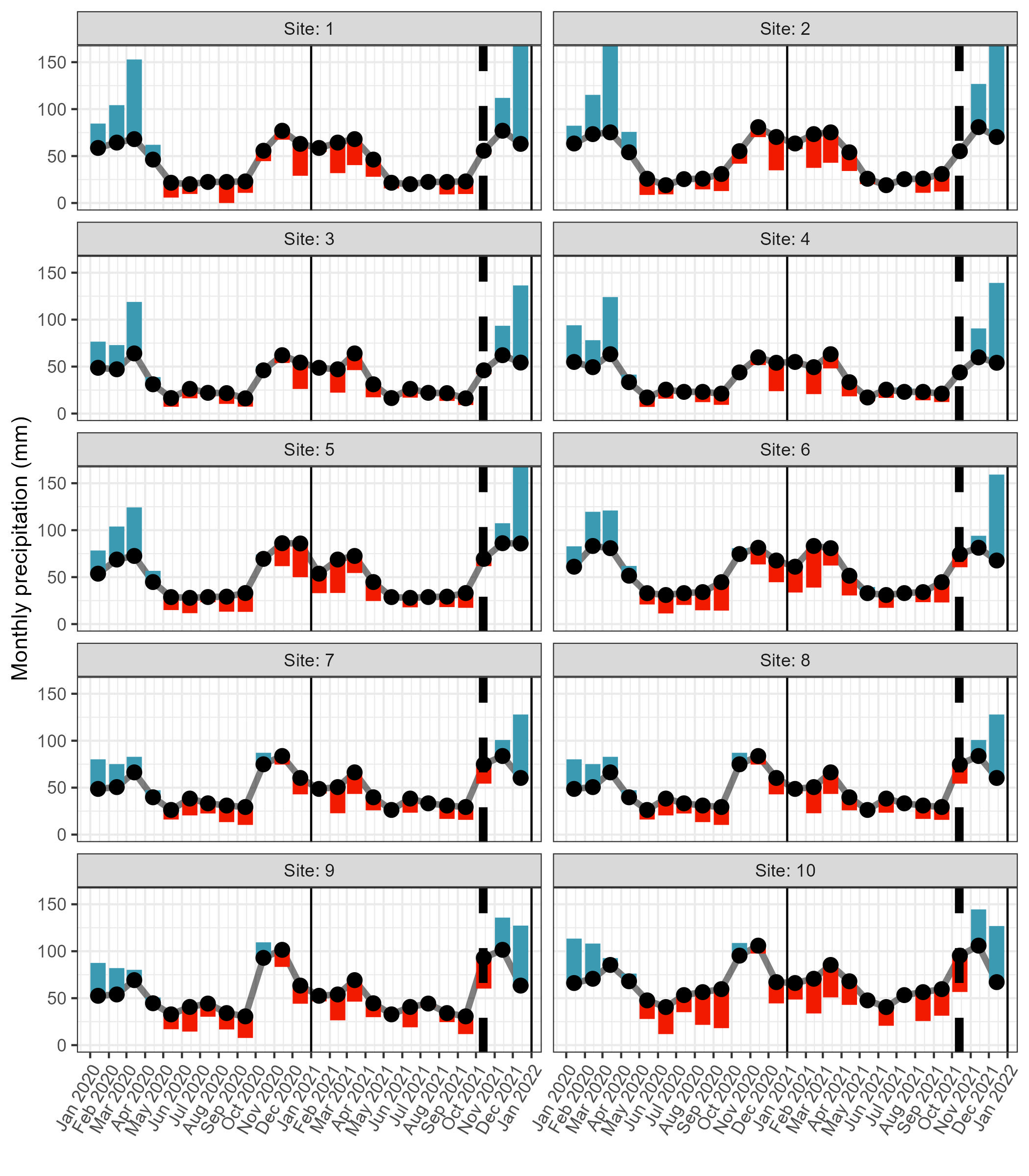

Supplement: Supplemental Information 4 — The monthly rainfall deviation from the mean monthly rainfall for ∼18 months prior to harvesting (13 October 2021: dashed line) of material from 10 populations for the experiment. Rainfall data was extracted from CHIRPS for each site, and mean monthly rainfall was calculated for the period 1981-01-01 to 2022-12-31. Note that we consider the CHIRPS estimated precipitation somewhat higher than actual in this region (unpublished weather station and rain gauge data). [file peerj-12-17471-s004.png]

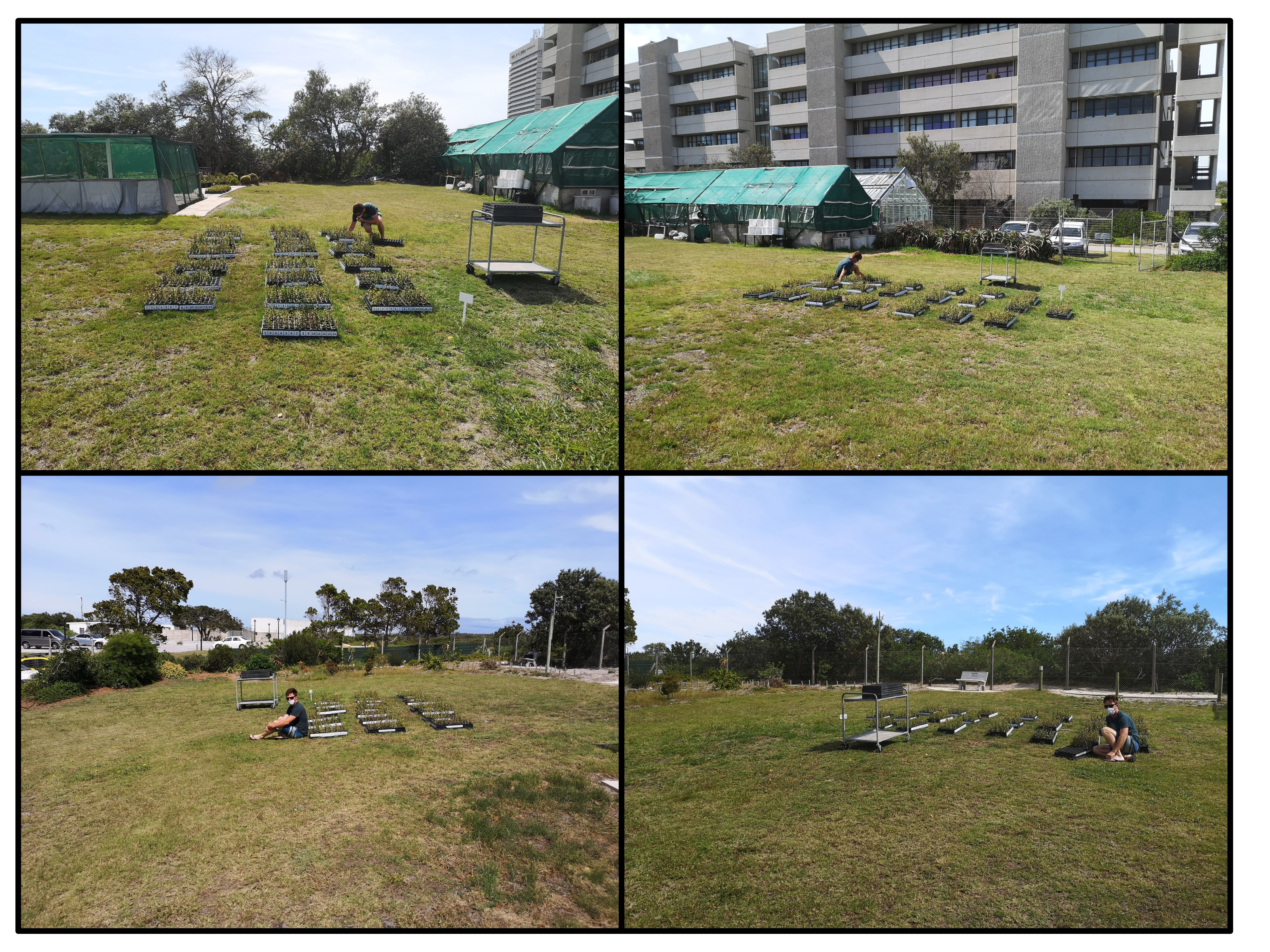

Supplement: Supplemental Information 5 [file peerj-12-17471-s005.png]

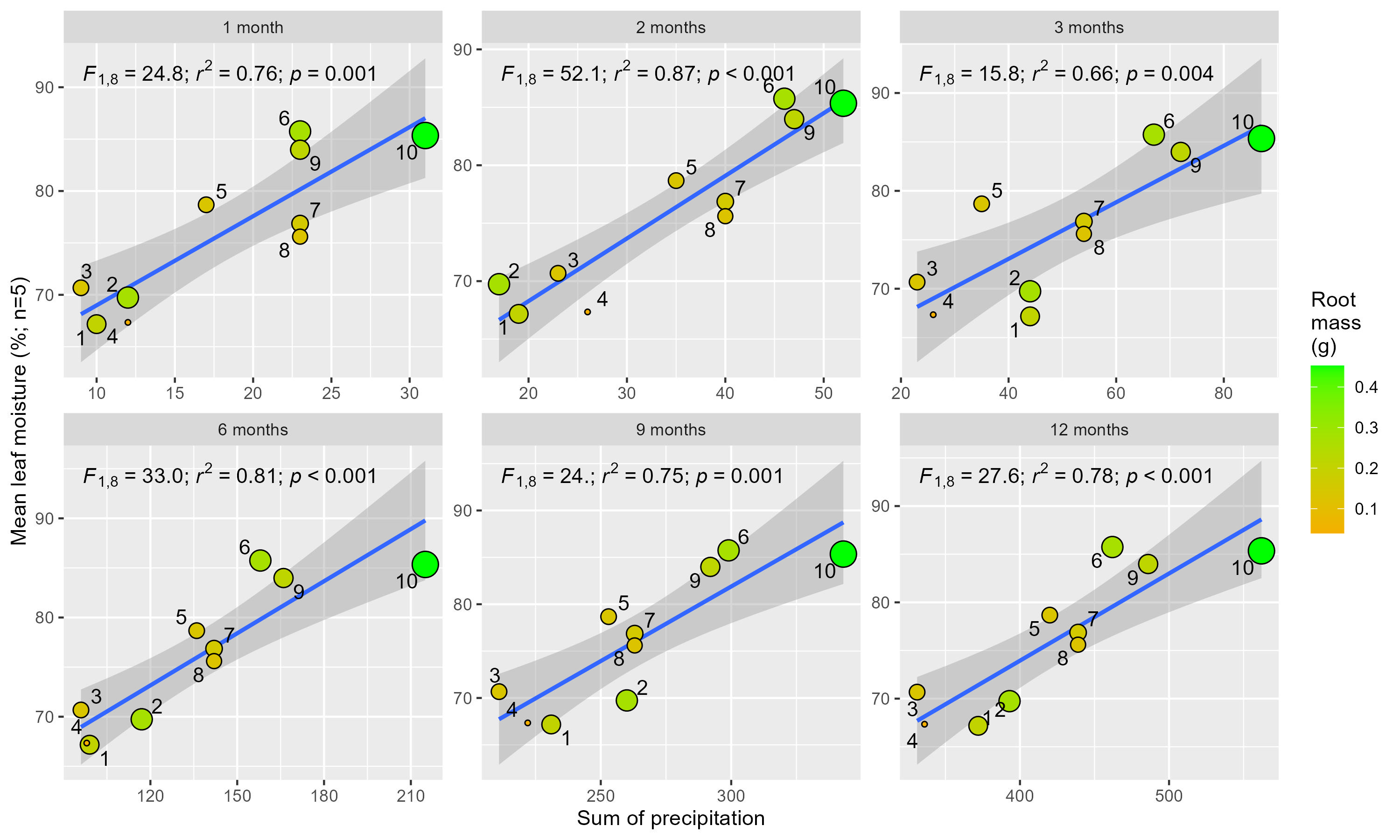

Supplement: Supplemental Information 6 — The precipitation values are from the CHIRPS dataset. We consider these satellite-derived estimated values higher than precipitation actually received in the field —e.g., a landowner’s rain gauge ∼3 km from population 8 only received 119 mm during the 12-month period prior to harvesting. Sites were selected from three landscape positions: inland mountains (1, 2, 5, 6), inland lowlands (3, 4, 7, 8) and coastal hills (9, 10). [file peerj-12-17471-s006.png]

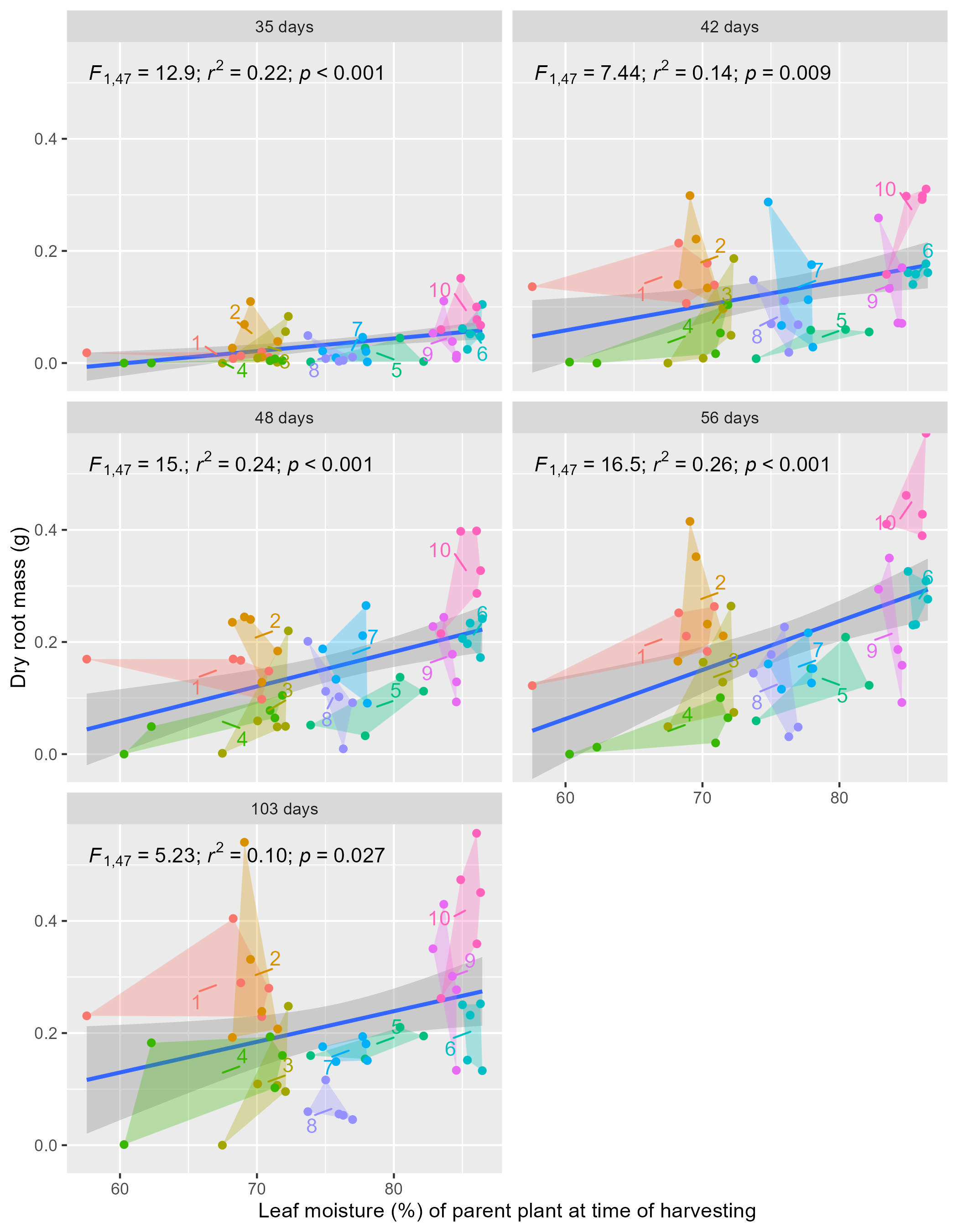

Supplement: Supplemental Information 7 — Different harvesting sites are represented by distinct colours, with numbers denoting the sites. Convex hulls highlight overall trends within and across sites. Linear regression analysis results are displayed in the top left of each panel. The harvesting sites originate from three landscape positions: inland mountains (1, 2, 5, 6), inland lowlands (3, 4, 7, 8), and coastal hills (9, 10). [file peerj-12-17471-s007.png]

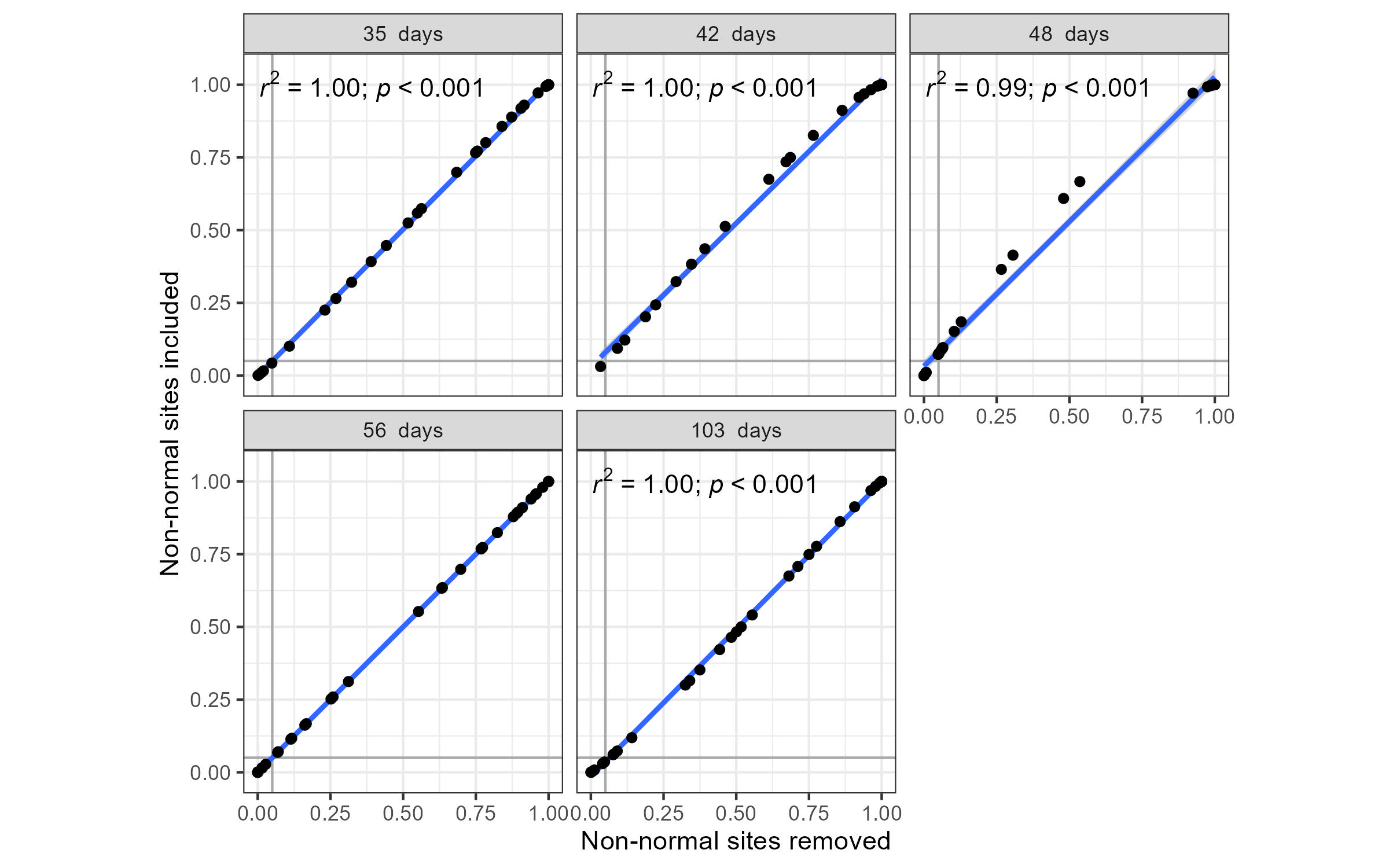

Supplement: Supplemental Information 8 — The five sampling events are different days since the start of the experiment. Note: No non-normal sites were identified at the 56-day sampling event, thus no linear regression model results are presented for this time point. [file peerj-12-17471-s008.png]
